# Prognostic factors of 10-year radiographic outcome in early rheumatoid arthritis: a prospective study

**DOI:** 10.1186/ar2498

**Published:** 2008-09-04

**Authors:** Natacha Courvoisier, Maxime Dougados, Alain Cantagrel, Philippe Goupille, Olivier Meyer, Jean Sibilia, Jean Pierre Daures, Bernard Combe

**Affiliations:** 1 Service de Rhumatologie, Hôpital Saint Antoine, 184 rue du Faubourg Saint Antoine, Paris, 75012, France; 2 Service de Rhumatologie B, Hôpital Cochin, 27 rue du Faubourg Saint Jacques, Paris, 75014, France; 3 Service de Rhumatologie, Hôpital Larrey, 24 chemin de Pouvourville, Toulouse, 31059, France; 4 Service de Rhumatologie, Hôpital Trousseau, avenue de la République, Tours, 37044, France; 5 Service de Rhumatologie, Hôpital Bichat, 46 rue Henri Huchard, Paris, 75018, France; 6 Service de Rhumatologie, Hôpital Hautepierre, 1 avenue Molière, Strasbourg, 67092, France; 7 Institut Universitaire de Recherche Clinique, 641 avenue du Doyen Gaston Giraud, Montpellier, 34093, France; 8 Service d'Immuno-Rhumatologie, Hôpital Lapeyronie, 371 avenue du Doyen Gaston Giraud, Montpellier, 34295, France

## Abstract

**Introduction:**

The objectives of this study were to determine the predictive factors of long-term radiographic outcome of rheumatoid arthritis (RA) and to describe the relationship between joint damage and disability over the course of the disease.

**Methods:**

A cohort of 191 patients with early RA referred from primary care physicians were prospectively followed for 10 years. To determine the predictive factors of radiographic outcome, univariate analysis of the relationship between baseline values and outcome measures was undertaken using a chi-squared or Fisher's exact test. Stepwise multiple logistic regression was also performed to select independent prognostic factors.

**Results:**

From data available for 112 patients, univariate analysis revealed a total Sharp score at 10 years that was significantly correlated with erythrocyte sedimentation rate (ESR), presence and level of IgA rheumatoid factor, presence of an anti-citrullinated protein antibody (ACPA), serum level of matrix metalloproteinase-3 and radiographic score at baseline. Logistic regression identified the baseline erosion score to be the most important baseline parameter as an independent prognostic factor of total radiographic score at 10 years (odds ratio = 5.64; 95% confidence interval = 1.78 to 17.86). After excluding radiographic scores from the entry parameters, the presence of ACPA and ESR were also predictive of the final total Sharp score. The Health Assessment Questionnaire (HAQ) score was strongly correlated with disease activity parameters, such as disease activity score and pain, at baseline and at three, five and 10 years. No correlation was found between total radiographic Sharp score and HAQ score throughout the study.

**Conclusions:**

In this prospective study, baseline radiographic score, ESR and ACPA were the best predictive factors of 10-year radiographic outcome in early RA. HAQ disability was associated with disease activity throughout the 10-year follow-up but not with joint damage. This discrepancy with previous reports may be due in part to the early start of therapy with disease-modifying anti-rheumatic drugs.

## Introduction

Rheumatoid arthritis (RA) is a potentially severe but heterogeneous disease. It can vary from mild to severe and in some cases can lead to severe joint damage and functional disability. Predicting RA outcomes is fundamental for optimal clinical management. Predictive factors of long-term outcome would help physicians determine the patients who will develop a severe form of the disease and treat them with appropriate aggressive therapy at an early stage. This ability is even more important with the availability of new treatments that can reduce or even stop the progression of RA. Radiographic damage is frequently used as a major assessment criterion for RA outcome. Numerous studies have identified possible initial individual factors associated with worse radiographic outcome, but there are many discrepancies between the studies and few were long-term (or) and prospective.

Joint damage increases slowly over the course of RA, and disability, decreasing during the first years, worsens with disease duration [1]. Disability in RA is influenced by parameters such as age, sex, social and psychological factors, muscle strength and co-morbidities. It is also associated with disease-related factors such as disease activity and joint destruction. The links between functional disability, joint damage and disease activity seem to vary with disease duration [2-4]. In early RA, functional impairment is believed to be mostly due to inflammatory processes as measured by disease activity [2,4-6]. In established RA, disability may be due to joint damage [2-4]. Prospective studies of the links between joint damage and functional disability are scarce and discordant in part, so the association between damage and disability remains uncertain.

Several assessment tools are available for measuring functional capacity. The easiest and cheapest are self-administered questionnaires. The most widely used instrument for assessing functional capacity in RA is the Health Assessment Questionnaire Disability Index (HAQ-DI) [7]. Joint damage is commonly assessed with radiographic scores, such as the Sharp score, modified by van der Heijde [8].

The main objective of our study was to determine the predictive factors of long-term radiographic outcome in early RA. The secondary objective was to describe the long-term outcome of joint destruction and disability in RA and their interrelation over the course of the disease.

## Materials and methods

### Patients

Between March 1993 and October 1994, all consecutive outpatients fulfilling the American College of Rheumatology criteria for RA for less than one year who had not been treated with disease-modifying antirheumatic drugs (DMARD) were referred to the study by primary care physicians from four French centres, Montpellier, Paris-Cochin, Toulouse and Tours. The patients had agreed to be enrolled in a 10-year follow up study, also giving signed informed consent. After inclusion, all patients were treated with DMARDs (methotrexate, sulfasalazine or both) that could be modified during the study according to efficacy and side effects. The study was approved by the ethical review board in Montpellier.

### Clinical assessment

The following data were collected by the same investigator for each patient at baseline and at three, five and 10 years: sex, age, disease duration (at baseline), pain on a visual analog scale (VAS), duration of morning stiffness, number of tender and swollen joints, disease activity score (DAS), presence or absence of nodules, and extra-articular manifestations.

### Biological assessment

Erythrocyte sedimentation rate (ESR) and C-reactive protein (CRP) levels were measured at baseline and at each follow-up visit in each centre. Measurements of the following were centralised in a single laboratory at baseline: immunoglobulin (Ig) A and IgM rheumatoid factor (RF) (anti-human Fc IgG ELISA), anti-keratin antibodies (indirect immunofluorescence [IF] on a cryostat section of rat oesophagus), anti-perinuclear antibodies (IF on buccal epithelial cells), anti-cyclic citrullinated peptide (anti-CCP) antibodies (ELISA kit; Immunoscan RA mark 2, Eurodiagnostica Arnhem, Netherlands), anti-nuclear antibodies (IF on Hep2 cells), anti-heat-shock protein 90 (anti-HSP90) antibodies (ELISA), YKL-40 (radioimmunoassay; Chondrex Metra Biosystems, Mountain view, CA, USA) and serum level of matrix metalloproteinase 3 (MMP 3) (ELISA) [9]. The patients who had positive results for anti-CCP, anti-keratin or anti-perinuclar antibodies were considered as ACPA (anti-citrullinated protein antibody) positive. Human leucocyte antigen (HLA) DRB1 and DQB1 genotyping was performed as previously described [10].

### Functional assessment

Functional disability of each patient was assessed by the HAQ at baseline and at three, five and 10 years [7]. This instrument has been adapted and validated in French [11]. Functional status of patients was scored on a continuous scale from 0 to 3. Patients were classified as mildly disabled (score < 1), moderately disabled (score 1 to 2) or severely disabled (score > 2) [12].

### Radiographic measurement

Patients underwent radiography of hands and feet in each centre at baseline and at three, five and 10 years. Radiographs were collated and evaluated blindly in chronological order according to the Sharp score modified by van der Heijde [8]. For each patient, an erosion score, a joint-narrowing score and a total radiographic score were noted. A single observer evaluated the radiographs at baseline and at 10 years. A second evaluation was performed on a random sample of 30 pairs of radiographs for validation. The intraclass correlation coefficient varied between 0.89 and 0.99. The radiographs taken at three and five years were evaluated by two other observers, who also performed a second evaluation on 30 pairs of radiographs taken at these same times. The intraclass and interobserver correlation coefficients were more than 0.85.

### Statistical methods

Statistical analysis was performed using SAS software, version 8-1 (SAS, Gary, IN). Outcome variables were dichotomised into qualitative variables: higher or lower than the median value for the total Sharp score at 10 years, and presence or absence of radiographic progression seen on radiography. Radiographic progression was defined by a change in the radiographic score greater than the 'minimum clinically important difference' (MCID). The OMERACT determined this MCID for the modified Sharp score to be five points [13].

Univariate analysis of the relation between all baseline values and outcome measures involved the chi-squared test or Fisher's exact test. Continuous variables were transformed into categorical variables with the median value used as the cut-off. A stepwise multiple logistic regression model was used to determine relevant independent prognostic variables. The prognostic variables included in the model were selected from results of the univariate analysis. The entry level was set at p = 0.10. Significant levels for changes over time in radiographic and HAQ scores were determined by the Friedman test. We used the MCID as determined by the OMERACT for the modified Sharp score. For the HAQ score, we used the MCID calculated by Kosinski of 0.24 points [14]. Comparative tests were performed to search for possible links between the variables studied over the time period: that is, between the HAQ and radiographic scores, and between the HAQ score and parameters of disease activity. We used the Wilcoxon test because the variables did not follow a normal distribution. Pearson's correlation coefficients were also calculated. The significance level was set at 0.05 for the whole study.

## Results

### Demographic, clinical and biological features of the patients

We enrolled 191 patients in the study, and 129 (67%) were followed up for 10 years. Forty-six patients were lost to follow-up, 11 died and five refused further follow-up. Radiographic data were available for 117 patients at 10 years (61.2%). Both HAQ score and radiographic data were available for 112 patients (58.6%).

Baseline characteristics of the 112 patients are shown in Table 1. No significant difference was found between the baseline characteristics of the 79 patients that were not available for the 10-year analysis and those of the 112 patients who were. Ninety (80.3%) patients were women; the mean (SD) age at diagnosis was 50.4 ± 12.6 years and the mean disease duration was 3.9 ± 2.8 months. Eleven (9.8%) patients presented with extra-articular manifestations. At baseline, DAS was 4.0 ± 0.7, ESR was 37.6 ± 26.7 mm and CRP level was 29.1 ± 39.8 mg/L. RF and anti-CCP antibodies were positive for 78.6% and 57.9% of patients, respectively. Seventy-eight (70.2%) patients had at least one RA-associated HLA-DRB1 allele.

**Table 1:** Baseline characteristics of patients (n = 112) with both radiographs and Health Assessment Questionnaire score available at 10 years

Characteristic	
Female	90 (80.3%)
Age (years)	50.4 ± 12.6
Disease duration (month)	3.9 ± 2.8
Pain on a VAS (mm)	54.8 ± 22.9
Morning stiffness (minutes)	90.1 ± 80.2
Swollen joints	8.9 ± 5.8
Ritchie index score	17.3 ± 8.5
DAS	4.0 ± 0.7
Extra-articular manifestations	11 (9.8%)
ESR (mm)	37.6 ± 26.7
CRP level (mg/L)	29.1 ± 39.8
IgA or IgM RF positivity	81 (78.6%)
Anti-CCP ab positivity	51 (57.9%)
HLA-DRB1*04 or 01 alleles	78 (70.2%)

Data are means ± standard deviation, unless otherwise indicated.

CCP, cyclic citrullinated peptide; CRP, C-reactive protein; DAS, disease activity score; ESR, erythrocyte sedimentation rate; HLA, human leucocyte antigen; RF, rheumatoid factor; VAS, visual analogue scale.

After inclusion, all patients received methotrexate, sulfasalazine or both. During the 10-year follow-up, patients received DMARDs for an average of 7.9 ± 3.3 years (Table 2). Methotrexate treatment was maintained for a mean length of 5.2 ± 3.2 years. Twenty-seven (24.1%) patients received therapy with a biological agent; 25 (22.3%) received one or more intra-articular corticosteroid or radionucleid injection. Twenty-six (23.2%) patients underwent joint surgery related to RA (32 procedures): 12 procedures were conservative treatments (synovectomy) and 20 were palliative (arthrodesis, arthroplasty or metatarsian resection). We could not get precise data regarding the use of oral corticosteroids over the 10-year period, because most of the patients received such drugs at various times and dosages. Nevertheless 33% of the patients who were evaluated after three years and 34.6% evaluated after five years had received a low dose of prednisone (5 to 15 mg/day) [15,16].

**Table 2:** Disease-modifying anti-rheumatic drugs (DMARDs) used during the 10 years of follow-up

DMARD	No (%) of patients	Mean time (months) ± SD
Methotrexate	86 (76.7%)	63.0 ± 39.2
Sulfasalazine	58 (51.7%)	44.7 ± 42.6
Methotrexate + sulfasalazine	33 (29.4%)	37.7 ± 33.9
Gold salts	33 (29.4%)	23.2 ± 24.0
Hydroxychloroquine	24 (21.4%)	20.6 ± 17.5
Leflunomide	24 (21.4%)	20.7 ± 18.2
D-penicillamine	2 (1.7%)	23.4 ± 23.2
Ciclosporin	4 (3.5%)	23.0 ± 23.3
Etanercept	12 (10.7%)	16.7 ± 18.3
Infliximab	9 (8.0%)	19.4 ± 7.0
Adalimumab	4 (3.5%)	15.4 ± 18.7
Anakinra	1 (0.8%)	8.9
Abatacept	1 (0.8%)	2.1

### HAQ disability

HAQ score decreased from 1.29 ± 0.71 at baseline to 0.53 ± 0.62 after three years, before a slow increase to 0.57 ± 0.62 and 0.75 ± 0.71 at five and 10 years, respectively, but never reaching the initial level (Table 3). At an individual level, most patients (80.4%) improved in functional score between the initial and final visits. A significant worsening (≥ MCID) was observed for only 14.1%.

**Table 3:** Radiographic and Health Assessment Questionnaire (HAQ) scores at baseline and after three, five and 10 years of follow-up (mean ± SD)

	Baseline n = 112	3 years n = 110	5 years n = 106	10 years n = 112
Erosion score	3.0 ± 5.7	4.8 ± 7.7	6.7 ± 9.9	18.4 ± 26.5
Joint narrowing score	2.7 ± 4.9	4.7 ± 8.6	10.5 ± 14.9	17.0 ± 21.2
Total Sharp score	5.8 ± 9.0	9.5 ± 14.9	17.3 ± 22.4	35.4 ± 46.1
HAQ score	1.29 ± 0.71	0.53 ± 0.62	0.57 ± 0.62	0.75 ± 0.71

### Radiographic outcome

Total radiographic Sharp score increased from 5.8 ± 9 at baseline to 9.5 ± 14.9, 17.3 ± 22.4 and 35.4 ± 46.1 at three, five and 10 years, respectively (Table 3). At baseline, 61 patients (54.4%) did not show any erosion compared with 19 patients (16.9%) at 10 years. Over the 10-year period, 69.6% of patients showed a significant progression in total radiographic score (≥ MCID). The average annual rate of increase in radiographic score was 2.96 points/year: 1.3 points/year between baseline and three years, 3.9 points/year between three and five years, and 3.62 points/year between five and 10 years.

### Predictive factors of radiographic outcome

In univariate analysis, the total radiographic Sharp score at 10 years was significantly correlated with the following baseline parameters (Table 4): ESR, positivity for and level of IgA RF; positivity for anti-perinuclear, anti-CCP and ACPA; serum level of MMP3; and radiographic scores (erosion score, joint narrowing score and total score). No significant correlation was found with any demographic, clinical (including joint count) or genetic data. The baseline parameters associated with erosion score and joint-narrowing score were similar (data not shown). Radiographic progression was significantly associated with age; positivity for and level of IgA RF; positivity for and level of IgM RF; positivity for anti-perinuclear, anti-keratin, anti-CCP and ACPA; serum level of MMP3; and radiographic scores (erosion score, narrowing score and total score).

**Table 4:** Baseline predictive factors of radiographic outcome at 10 years (univariate analysis)

**Baseline variables**	Total Sharp score at 10 years		Radiographic progression	
	
	p	OR (95% CI)	p	OR (95% CI)
Total Sharp score	< 0.0001	8.66 (3.72–20.15)	0.003	3.72 (1.51–9.15)
Erosion score	< 0.0001	6.00 (2.68–13.39)	< 0.0001	8.47 (2.98–24.05)
Joint narrowing score	< 0.0001	4.71 (2.14–10.34)	0.007	3.18 (1.33–7.62)
ACPA	0.002	3.91 (1.59–9.61)	< 0.0001	10.88 (4.03–29.3)
Anti-CCP antibodies	0.005	3.37 (1.40–8.11)	< 0.0001	8.62 (2.97–25.00)
IgA RF positivity	0.01	3.19 (1.28–7.91)	0.0001	5.89 (2.27–15.28)
ESR	0.01	2.55 (1.21–5.39)	0.34	1.47 (0.66–3.26)
IgA RF level	0.02	2.40 (1.10–5.21)	0.004	3.86 (1.46–10.17)
MMP3 level	0.03	2.60 (1.07–6.30)	0.003	5.00 (1.61–15.50)
Anti-perinuclear antibodies	0.03	2.28 (1.05–4.93)	0.0002	6.11 (2.22–16.77)
Swollen joint count	0.05	0.48 (0.23–1.00)	0.88	1.05 (0.47–2.33)
Morning stiffness	0.08	2.10 (0.90–4.87)	0.31	1.65 (0.61–4.42)
HLA-DRB1*01	0.08	0.49 (0.22–1.10)	0.07	0.47 (0.20–1.08)
Tender joint count	0.08	0.48 (0.21–1.10)	0.13	0.48 (0.18–1.26)
IgM RF positivity	0.10	1.95 (0.87–4.39)	0.001	4.08 (1.63–10.20)
CRP	0.19	1.63 (0.77–3.42)	0.49	1.32 (0.58–2.99)
DAS	0.26	1.52 (0.73–3.16)	0.44	1.36 (0.61–3.05)
Anti-keratin antibodies	0.32	1.46 (0.68–3.12)	0.02	2.76 (1.09–7.00)
Age	0.34	1.42 (0.67–3.00)	0.007	3.28 (1.35–7.98)
Sex	0.40	0.67 (0.27–1.69)	0.14	0.42 (0.13–1.36)
Pain on VAS	0.45	1.36 (0.60–3.07)	0.44	0.69 (0.27–1.77)
YKL 40	0.56	1.69 (0.27–10.59)	0.36	2.30 (0.36–14.66)
Anti-nuclear antibodies	0.64	0.81 (0.34–1.93)	0.79	1.13 (0.42–3.01)
Extra-articular signs	0.68	1.29 (0.37–4.50)	0.84	1.15 (0.28–4.63)
IgM RF level	0.76	1.12 (0.52–2.39)	0.01	3.05 (1.19–7.79)
Ritchie score	0.85	1.06 (0.51–2.21)	0.86	1.07 (0.48–2.38)
Anti-HSP90 antibodies	0.92	0.95 (0.37–2.43)	0.21	0.53 (0.19–1.44)
HAQ score	0.95	0.97 (0.43–2.19)	0.60	0.77 (0.30–2.00)

ACPA, anti-citrullinated protein antibody; CCP, cyclic citrullinated peptide; CI, confidence interval; CRP, C-reactive protein; DAS, disease activity score; ESR, erythrocyte sedimentation rate; HAQ, Health Assessment Questionnaire; HLA, human leucocyte antigen; HSP90, heat shock protein 90; MMP3, matrix metalloproteinase 3; OR, odds ratio; RF, rheumatoid factor; VAS, visual analogue score.

The most important baseline parameter identified by logistic regression as an independent predictive factor of total radiographic Sharp score at 10 years was erosion score (odds ratio [OR] = 5.64; 95% confidence interval [CI] = 1.78 to 17.86) (Table 5). After excluding radiographic scores from the entry parameters, the presence of ACPA and ESR were also predictive of the final total Sharp score. The final erosion score was predicted by the baseline erosion (OR = 7.33; 95% CI = 2.13 to 25.26) and joint narrowing scores (OR = 3.73 and 95% CI = 1.09 to 12.73) and, after excluding radiographic scores from the entry parameters, by the presence of ACPA (OR = 4.22; 95% CI = 1.30 to 13.65). The joint narrowing score was predicted by the baseline erosion score (OR = 8.98; 95% CI = 2.67 to 30.15).

**Table 5:** Stepwise logistic regression analysis of predictive factors of total Sharp score at 10 years

	Coefficient	SE	OR	95% CI
Constant	-1.085	0.513		
Erosion score	1.729	0.588	5.64	1.78–17.86
*After excluding the baseline radiographic scores from the model:*				
Constant	-1.345	0.631		
ACPA	1.353	0.631	3.87	1.17–12.75
ESR	1.166	0.514	3.20	1.17–8.78

ACPA, anti-citrullinated protein antibody; CI, confidence interval; ESR, erythrocyte sedimentation rate; SE, standard error; OR, odds ratio.

### Relation between HAQ score, radiographic scores and disease activity parameters

Table 6 shows HAQ scores strongly associated with disease activity parameters such as DAS, pain and Ritchie score at baseline and at three, five and 10 years (p < 0.0001). No correlation was found between total Sharp score and HAQ score throughout the study. Only erosion score was weakly but significantly associated with HAQ score at five years (r = 0.16; p = 0.007).

**Table 6:** Correlations between Health Assessment Questionnaire score and disease activity parameters and radiographic scores at baseline and at three, five and 10 years of follow-up

	Baseline		3 years		5 years		10 years	
	
	p	r	p	r	p	r	P	r
DAS	< 0.0001	0.56	< 0.0001	0.57	< 0.0001	0.58	0.0001	0.41
Pain on VAS	0.001	0.43	< 0.0001	0.69	< 0.0001	0.60	< 0.0001	0.57
Morning stiffness	0.06	0.38	< 0.0001	0.60	< 0.0001	0.55	< 0.0001	0.46
Swollen joints	0.03	0.16	0.001	0.19	< 0.0001	0.34	0.48	0.09
Ritchie index score	< 0.0001	0.47	< 0.0001	0.57	< 0.0001	0.56	< 0.0001	0.46
ESR	0.10	0.24	0.04	0.27	0.006	0.29	0.05	0.17
CRP	0.0004	0.35	0.0004	0.25	0.02	0.11	0.69	0.04
Erosion score	0.78	0.01	0.74	0.08	0.007	0.16	0.46	0.14
Narrowing score	0.76	0.06	0.80	0.24	0.39	0.10	0.64	0.17
Total Sharp score	0.51	0.04	0.86	0.18	0.06	0.14	0.50	0.16

CRP, C-reactive protein; DAS, disease activity score; ESR, erythrocyte sedimentation rate; VAS, visual analogue score

## Discussion

Evaluating the prognosis of RA is more than ever of high importance. Many studies have been published on this subject, but the results are often conflicting. Discrepancies are probably due to differences between study designs and length of follow-up. Only five prospective studies were conducted over more than seven years [17-21]. We performed a long-term study of 10 years to investigate predictive factors of radiographic outcome in RA and found the best independent predictive factor of the 10-year radiographic score to be baseline erosion score. After excluding radiographic scores from the entry parameters, the presence of ACPA and ESR were also predictive of the final total Sharp score.

The predictive value of the baseline radiographic score has been shown in many short-term studies [15,22-24] and two long-term studies [17,18]. For Kaarela and colleagues, who followed 200 patients for six to nine years (mean 7.6 years), the independent predictive factor of final radiographic score was baseline radiographic score [17]. In the study by Lindqvist, the baseline radiographic score, determined by the Larsen's method, was correlated with radiographic progression at five and 10 years by univariate analysis [18]. In the other long-term cohorts, the predictive value of the initial joint destruction was not studied.

In our study, the presence of anti-CCP antibodies at baseline was strongly associated with total radiographic score after 10 years, but this parameter was not selected as an independent predictive factor on multivariate analysis, because of the number of missing data (24 of 112) at baseline. With the contribution of data on anti-perinuclear or anti-keratin antibodies, the logistic regression model identified the presence of ACPA at baseline as an independent predictive factor of the total Sharp score after 10 years. The predictive value of the anti-CCP antibodies had already been suggested in short-term studies [22,24-26]. Also, in a recent 10-year longitudinal study, Syversen and colleagues showed the presence and level of anti-CCP antibodies was predictive of radiographic progression [20].

A high ESR at baseline also predicted an elevated final total Sharp score in our study. This result is in agreement with that of most short-term [15,23,27,28] and long-term studies [17,20,21]. In our study, CRP level was not predictive of the final Sharp score, and the predictive value of this parameter remains controversial in the literature, perhaps because of different dosage techniques.

Being positive for and the level of IgA RF present were strongly correlated to 10-year Sharp score by univariate analysis, but these parameters were not selected as independent predictive factors on logistic regression. Both IgA and IgM RF were also correlated with radiographic progression. Very few studies have distinguished the predictive value of these two isotypes of RF. Syversen and colleagues showed IgM RF was an independent predictive factor of 10-year radiographic progression. Lindqvist and colleagues obtained an equivalent result during the same length of follow-up but did not distinguish between IgA and IgM RF in one of their studies [18]. In another study, they showed a significant association between the presence of IgA RF and more severe joint damage after five years, but the presence of RF, whatever the isotype, did not predict the radiographic score after 10 years [19]. Two other long-term studies also isolated the presence of RF at baseline as an independent predictive factor of radiographic score after 7.6 and 8.6 years [17,21].

We also noticed the potential interest of the MMP3 level, involved in degradation of cartilage proteoglycans, as a predictive factor of radiographic outcome. We found quite a strong correlation between baseline level of MMP3 and final radiographic score and with 10-year radiographic progression. To our knowledge, ours is the only long-term study to take this baseline parameter into account. Two short-term studies had shown such a correlation [29,30].

The role of several demographic and clinical parameters, such as sex, age and number of tender or swollen joints, has been suggested by several short-term studies, with conflicting results. None of these factors were shown to be independent prognostic factors in our study. In long-term studies, only Syversen and colleagues found an influence of the female sex on prognosis [20]; Kaarela and colleagues found elevated age at diagnosis and a high number of swollen joints to be independent prognostic factors [17], but these findings were not confirmed in other studies.

The predictive value of the presence of the shared epitope, suggested in our study after three years of follow-up and in a few other short-term studies, was not confirmed after 10 years [15,31-33]. These results are in agreement with Lindqvist and colleagues who found the presence of the shared epitope to be predictive of radiographic progression during the first five years but not after five years of follow-up [18]. Even if the decrease in the number of patients with time can partly explain these results, we can assume that the genetic data influence the radiographic outcome in the short but not long term.

As mentioned in the results, in this study it was unfortunately not possible to carefully analyse the potential effect of corticosteroids during the first years of the disease on the occurrence of erosions.

The mean HAQ score at baseline was higher in our cohort than that found in other prospective studies of early RA (1.29 vs. 0.63 to 1) [2,34,35]. This result may be due to our patients not having received DMARDs at baseline. The decrease in HAQ score we observed at three years confirmed the results of two previous studies showing an improvement in functional capacity during the first two years of RA [36,37]. After 10 years, the mean HAQ score for our patients was similar to that from other cohorts.

The radiographic scores in our study were slightly lower than those for other prospective cohorts at baseline: the median total modified Sharp score reached only 2 in our cohort compared with 11 in the Welsing and colleagues study and 12 in the Drossaers-Bakker and colleagues study [2,3]. However, the proportion of patients with radiographic erosion at baseline was similar to that found in the study by Lindqvist and colleagues [18]. Of interest, after 10 years, the radiographic score in our study was very low compared with that for other cohorts: the median reached only 18 in our study compared with 83 in the Welsing and colleagues study after nine years and 145 in the Drossaers-Bakker and colleagues study after 12 years [2,3]. The proportion of patients with no erosion at 10 years was 16.9% in our study compared with 4% in the Lindqvist and colleagues cohort [18]. In our study, joint damage gradually, slightly worsened over the 10-year follow-up, without a higher progression rate during the first years of the disease as shown in previous reports [38,39]. The most likely explanation is the difference in treatments received by patients, because in our study most patients were treated with DMARDs, such as methotrexate, which have demonstrated a structural effect, compared with older studies where patients received other DMARDs, frequently hydroxychloroquine.

We did not find any correlation between HAQ and Sharp scores throughout the study, but disease activity and radiographic scores remained strongly linked. Several previous studies had suggested that functional capacity was influenced largely by disease activity in early RA and by joint destruction in established RA [2-4]. However, the radiographic scores we found after 10 years were lower than those observed in these older studies. Our results could reflect the consequences of an adequate management of early RA, and perhaps the expected links between HAQ and Sharp scores appear much later in the evolution of the disease.

## Conclusion

In this prospective study, baseline radiographic score, ESR and ACPA were the best predictive factors of 10-year radiographic outcome in early RA. HAQ disability was associated with disease activity throughout the 10-year follow-up but not with joint damage. This discrepancy with previous reports may be partly due to the early start of DMARD therapy.

## Abbreviations

ACPA: anti-citrullinated protein antibody; CCP: cyclic citrullinated peptide; CI: confidence interval; CRP: C-reactive protein; DAS: disease activity score; DMARD: disease-modifying antirheumatic drugs; ESR: erythrocyte sedimentation rate; HAQ-DI: Health Assessment Questionnaire Disease Index; HLA: human leucocyte antigen; HSP90: heat-shock protein 90; IF: immunofluorescence; Ig: immunoglobulin; MCID: minimum clinically important difference; MMP3: matrix metalloproteinase 3; OR: odds ratio; r: Pearson's correlation coefficient; RA: rheumatoid arthritis; RF: rheumatoid factor; SD: standard deviation; SE: standard error; VAS: visual analog scale.

## Competing interests

The authors declare that they have no competing interests.

## Authors' contributions

NC participated in acquisition, analysis and interpretation of data and drafted the manuscript. MD, AC and PG participated in the design of the study and acquisition of data. OM and JS carried out the immunoassays. JPD participated in the design of the study and performed the statistical analysis. BC conceived the study, participated in its design, analysis and co-ordination and helped to draft the manuscript. All authors read and approved the final manuscript.
